# Migrant older adults’ perception of social support on subjective well-being: a mediating role of psychological resilience

**DOI:** 10.3389/fpubh.2025.1647544

**Published:** 2025-10-01

**Authors:** Yuxi Liu, Qian Liu, Qihui Gan, Li Jia, Xianglei Zhu, Ruiming Liu, Jie Huang, Chonghua Wan, Qikang Chen

**Affiliations:** ^1^Shunde Women and Children’s Hospital, Guangdong Medical University, Foshan, China; ^2^School of Humanities and Management, Health Law and Policy Institute, Guangdong Medical University, Dongguan, China

**Keywords:** migrant older adults, perception of social support, SWB, resilience, mediating effect

## Abstract

**Introduction:**

With the acceleration of urbanisation in China, there are a growing number of migrant older adults, and the mental health problems of this group require prompt attention.

**Methods:**

This research conducted a questionnaire survey of 470 migrant older adults in various communities of Dongguan, and employed an independent sample t-test, Pearson correlation analysis, and stepwise multiple regression to analyse the data to explore the relationship between perceived social support, resilience and subjective well-being (SWB).

**Results:**

The findings indicated that migrant older adults’ perceived social support is positively correlated with resilience (r = 0.827, *p* < 0.05) and SWB (r = 0.645, *p* < 0.05), and resilience and SWB are also positively correlated (r = 0.698, *p* < 0.05). The SWB of migrant older adults can be predicted from their perceived social support (*β* = 0.182, *p* < 0.05) and resilience (β = 0.281, *p* < 0.05). Moreover, resilience partially plays a mediating role between the perception of social support and SWB, and the mediating effect accounts for 60.8 percent of the total effect.

**Discussion:**

To improve the SWB of the migrant older adults, it is important to address the psychological potential of the migrant older adults from the perspective of positive psychology, and help them improve their positive psychological quality and resilience.

## 1 Introduction

With the acceleration of urbanisation in China, a growing number of workers and their families from across the country have migrated to work and live in cities, especially those with a high economic status such as the Beijing, Shanghai and Guangzhou where jobs are more plentiful and lucrative (1, 2). Corresponding with the filial piety as a major Chinese traditional value, the Chinese older adults family members are brought along with their children who migrate for work to new cities with the responsibility to take care of their parents. Other reasons for the migration of the older adults parents are to take care of their grandchildren and to reunite with their families (3). In China, the internal, migrant older adult is a particular product of China’s social transformation period. It is also a unique phenomenon in the aging process of China with its specific characteristics (3). Leaving their homeland for reasons such as caring for their adult children or grandchildren, these migrant older adults must adapt to the urban environment at an old age (2, 3). Thus, they will inevitably face challenges in their daily lives, such as pastimes and communication, bringing negative emotions that affect their physical and mental health. As the migrant older adults face various problems adapting to society, they need special attention.

In recent years, some scholars have explored the social adaptation process of migrant older adults from the perspective of social work (4, 5). They have attempted to address the dilemma of the migrant older adult’s urban adaptation through casework, group counselling, community intervention, and other means (4, 5). However, most existing research on the Chinese migrant older adult group primarily discusses the problems encountered in urban adaptation and the effective ways to solve them from a negative perspective, few focus on the psychological aspects of the migrant older adult from a positive perspective and explore this group in depth from a psychological perspective (6, 7). Furthermore, there are few empirical studies on the migrant older adult in China. Thus, this paper focuses on the SWB of the migrant older adult from a positive psychology perspective. Using empirical research methods, the present study explores the relationship between this group’s SWB, perceived social support, and psychological resilience to provide a theoretical basis for enhancing the migrant older adults’ quality of life and their physical and mental health.

### 1.1 ‘Subjective well-being’ and its influencing factors

The term ‘subjective well-being’ originated from positive and health psychology. It is an indicator for individuals to describe their level of well-being according to their subjective evaluation of life, including judgement of life satisfaction and positive or negative subjective feelings (8, 9). It has aroused people’s constant concerns about their mental health. Researchers generally believe that SWB includes two aspects, namely, life satisfaction—the individual’s evaluation of their quality of life and satisfaction, and emotional experience—including positive and negative emotional experiences (9). Research demonstrates that SWB is an important comprehensive psychological indicator to measure the older adults’s mental health and personal quality of life (10, 11). Improving SWB can effectively promote the healthy development of individual psychology, work, and social relations, thereby significantly improving quality of life (9).

Some researchers indicate that the factors that affect the SWB of the older adults are primarily, objective or subjective (7, 11). According to the existing literature, the objective factors include gender, age, economic conditions, religious traditions, family environment, and other aspects. Marital status and the marital relationship also affect the older adults’s SWB. Compared with the older adults with spouses, widowed older adults have lower SWB and relatively less social support (3). For the older adults with spouses, their marital emotional status predicts their SWB. Other predictors include intergenerational economic assistance, meeting frequency, and emotional communication (1, 7). The impact of living with children on the well-being of the older adults depends on the family type (2, 4). For instance, the older adults without a spouse living with children have increased well-being. In contrast, the opposite effect for the older adults with a spouse is observed. In addition, the factors that affect the SWB of the older adults include personality, self-efficacy, attitudes towards themselves and the environment. For example, studies have affirmed that the personality and general self-efficacy of the older adults could predict their SWB (12, 13). In addition, the older adults’s self-rated physical health significantly positively correlated with their satisfaction with children and SWB (13, 14). A number of existing studies explored the migrant older adult’s SWB, but most proposed ways to improve it in the light of social work. However, few studies thoroughly touched on the current state of their SWB and the factors that affect it.

### 1.2 Social support and related research

Social support refers to the resources individuals obtain from social activities, including material or spiritual help from family and friends (15). There are two categories of social support-objective and subjective or actual and perceived. Objective or actual social support includes material assistance and direct services to individuals. Subjective or perceived social support refers to the emotion an individual experiences when he or she feels respected, understood, and supported. Social support can act as a buffer when individuals experience negative events; it is one of the most critical factors influencing individuals’ adaptation to social situations (16, 17).

Unfortunately, research indicates that social support for the older adults is declining significantly, with some scholars identifying deficiencies in financial, caregiving, and spiritual and emotional support for the migrant older adults (18). However, social support can influence the quality of life of the migrant older adults and their psychological well-being-older people with higher levels of social support have higher levels of psychological well-being (17, 18). For example, a study of the social support networks and integration of migrant older adults revealed that the size and quality of their social support network positively predicted their life satisfaction (19). Therefore, this paper focuses on the effect of perceived social support on the SWB of the migrant older adults.

### 1.3 Psychological resilience and its related research

Psychological resilience, or ‘mental toughness’ is the ability that helps individuals to recover from adverse experiences and adapt to the environment. It is a static and stable psychological resource that belongs to individuals, protecting them from dangerous factors (20, 21). More specifically, resilience is the ability of individuals to actively use their internal and external resources to adapt to the environment when facing pressure or adversity.

Research indicates that the psychological resilience of the older adults is closely related to depression and health status. Furthermore, it finds a significant negative correlation between the older adults’s resilience, depression, and high levels of negative life events (22, 23). More specifically, the higher the level of the older adults’s resilience, the lower the frequency at which depression and negative life events occur. In addition, research has discovered a positive correlation between the older adults’s social support, psychological resilience, self-esteem, and various health status factors (24). As such, social support positively predicts psychological resilience, with self-esteem having a significant mediating effect.

Moreover, psychological resilience, a psychological resource to buffer individuals from stress, is a mediating moderator. Research examining the relationship between psychological resilience and stress, using physical symptoms, anxiety, worry, and depression/family relationships as mental health factors, has discovered that psychological resilience is negatively related to psychological health and stress (24). Amongst the findings, psychological pressure and resilience are predictive of mental health, and resilience mediates the relationship between psychological pressure and mental health (25).

### 1.4 Relationship between social support, resilience and SWB

Existing studies show a close relationship between social support, psychological resilience, and SWB. There is a significant correlation between resilience, social support, and two dimensions of SWB, namely, life satisfaction and positive/negative emotions (26, 27). Perceived social support is significantly predictive of SWB (28, 29). The higher the perceived social support, the higher the SWB. In addition, a significant positive correlation exists between resilience and the SWB of the older adults (30, 31). The older adults with high resilience have higher SWB than those with low- and medium-level resilience. Some studies have also discovered a positive relationship between the social support of the older adults and resilience (32, 33). The higher the level of social support, the higher their resilience. Moreover, the older adults’s perceived social support can affect their resilience, reducing their risk of depression (33). According to some studies, psychological resilience plays a significant role in partially mediating the relationship between social support and SWB (33, 34).

**Figure 1:**
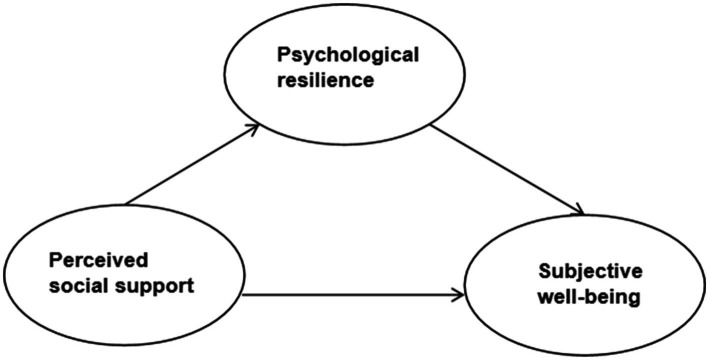
Relationship model of perceived social support, mental resilience and subjective well-being.

In summary, this paper proposes the following hypotheses (see Figure 1): The migrant older adults perceived social support will positively and significantly predict their SWB; and psychological resilience of the migrant older adults plays a significant mediating role in perceiving social support and predicting SWB.

## 2 Materials and methods

### 2.1 Participants

A community-based questionnaire survey was conducted in Dongguan city, in South China, from December 2018 to December 2019. Participants inclusion criteria in this research were migrant older adults aged 60 or more years, who had come at least 6 months prior to the study and were not listed in the Dongguan household registration system. The eligible list of migrant older adults for the present study was provided by the community committee. To identify the participants, a multi-stage cluster sampling survey technique was applied. Ultimately, 470 migrant older adults participated in this study.

### 2.2 Measurements

#### 2.2.1 Perceived social support scale

The Perceived Social Support Scale (PSSS) measures social support in the present study. The scale measures three dimensions, namely, family support, friend support, and other support. It uses a seven-point scoring scale with 12 self-rated items. The higher the score of the items on dimensions and overall scale, the higher the level of perceived social support. In existing studies, the scale’s Cronbach’s alpha was above 0.8 (35). In this research, Cronbach’s alpha coefficient in the overall scale was 0.913.

#### 2.2.2 Ego resilience scale

This research measured resilience with the Ego Resilience Scale, compiled by Block and Kremen (36). It scores on a four-point scale with 14 items. The higher the score of the items on the scale, the better the resilience. In this research, the scale’s Cronbach’s alpha coefficient was 0.881 (36).

#### 2.2.3 Subjective well-being

SWB was assessed by the Memorial University of Newfoundland Scale of Happiness (MUNSH), and has high validity [Kaiser-Meyer-Olkin (KMO) of 0.703] and consistency (Cronbach’s alpha of 0.735) (37). The MUNSH is a multi-item scale which has 24 items, assessing four dimensions, namely, positive emotion (PA), general positive experience (PE), negative emotion (NA), and general negative experience (NE). Numerous items on this scale cover specific content in the geriatric area with reference to age and time of life. Total scores range between −24 to + 24 points, where higher scores indicate better SWB (37).

### 2.3 Data collection

The research team visited the study areas to initiate contact with the community leaders to gain some information from their community and households. The questionnaires were tested in a pilot study. Face-to-face interviews using the structured questionnaire were conducted. All of the participants were interviewed at their home using their local language by trained interviewers. Each interview took approximately 20 to 25 min.

### 2.4 Statistical analysis

Epi-data version 3.1 was used for quantitative data entry. Double data entry and validation were conducted. The analyses were based on complete case analysis, which mean only the participants without missing data were included. All analyses were conducted using R version 3.4.2. All *p*-values were two-tailed and statistical significance level was set at less than 0.05.

## 3 Results

Table 1 indicated there was a total of 470 migrant older adults, the mean age was 67.5 ± 5.5 years and 47.9 percent were men. Amongst these older adults, 97.4 percent are Han Chinese, with the highest number of migrant older adults from Hunan province at 23.4 percent, followed by Guangdong province at 21.9 percent. 75.3 percent of the migrant older adults have an education level of junior high school or below; 79.8 percent of migrant older adults are married, whilst 20.2 percent are divorced or widowed. 77.2 percent of migrant older adults’ households have a population of four to six people. For 62.1 percent of them, income comes from their sons or daughter. A majority of these older adults who move with them take care of their children and their offspring.

**Table 1:** General characteristics of the participants sample (*N* = 470).

Categories	Groups	*n* (%)
Ethnicity	Han ethnicity	458 (97.4)
	Minority ethnicities	12 (2.6)
Place of Birth	Hunan province	110 (23.4)
	Guangdong province	103 (21.9)
	Other province	257 (54.7)
Education level	Primary school	163 (34.7)
	Junior high school	191 (40.6)
	High school	83 (17.7)
	Junior college	16 (3.4)
	Above an undergraduate degree	17 (3.6)
Gender	Male	225 (47.9)
Marital status	Married	375 (79.8)
	Divorced or widowed	95 (20.2)
Family population	≤3 persons	35 (7.5)
	4–6 persons	363 (77.2)
	7–9 persons	72 (15.3)
Source of income	Retirement pay	132 (28.1)
	Spouse	11 (2.3)
	Sons or daughter	292 (62.1)
	Social assistance	5 (1.1)
	Others	30 (6.4)
Age	67.5 ± 5.5	

Firstly, Pearson correlation analysis was conducted on the perceived social support, psychological resilience, and SWB of the migrant older adults, and it was discovered that there were positive correlations between the three factors. There was a significant positive high correlation between perceived social support and psychological resilience, and a significant positive moderate correlation between SWB and perceived social support and psychological resilience (correlation coefficients are shown in Table 2).

**Table 2:** Correlations Between psychological resilience, perceived social support and SWB.

Variable	Psychological resilience	Perceived social support	SWB
Psychological resilience	1		
Perceived social support	0.827^***^	1	
SWB	0.698^***^	0.645^***^	1

*p < 0.05; **p < 0.01; ***p < 0.001.

Thereafter, regression analysis was conducted on the perceived social support and psychological resilience of the migrant older adults using their SWB and various dimensions of SWB (positive emotion, general positive experience, negative emotion, and general negative experience) as outcome variables. The results indicate (see Table 3) that the perceived social support and psychological resilience of the migrant older adults have significant predictive effects on SWB and its various dimensions (*p* < 0.01).

**Table 3:** Regression analysis of perceived social support on SWB.

Dependent variable	Predictive variable	β	t	R^2^	F
SWB	Perceived social support	0.182	16.15^***^	0.389	263.32^***^
	Psychological resilience	0.281	18.11^***^	0.452	328.95^***^
Positive emotion	Perceived social support	0.065	15.76^***^	0.364	247.68^***^
	Psychological resilience	0.089	15.63^***^	0.360	243.75^***^
Positive experience	Perceived social support	0.059	12.35^***^	0.265	153.32^***^
	Psychological resilience	0.087	13.54^***^	0.296	182.49^***^
Negative emotion	Perceived social support	−0.021	−3.68^***^	0.030	13.21^***^
	Psychological resilience	−0.043	−5.69^***^	0.069	32.08^**^
Negative experience	Perceived social support	−0.018	−2.99^***^	0.027	12.26^***^
	Psychological resilience	−0.039	−5.02^***^	0.065	31.52^**^

*p < 0.05; **p < 0.01; ***p < 0.001.

Finally, using stepwise multiple regression analysis, a mediation effect analysis was conducted with psychological resilience as the mediating variable, perceived social support as the independent variable, and SWB as the dependent variable. The results revealed (see Table 4) that with SWB as the dependent variable and perceived social support as the independent variable, the regression coefficient had statistical significance (β = 0.186, *p* < 0.001).

**Table 4:** The mediating effect of psychological resilience on perceived social support and SWB.

Model	Dependent variable	Independent variable	β	t	R^2^	F
1	SWB	Perceived social support	0.186	16.33^***^	0.379	263.58^***^
2	Psychological resilience	Perceived social support	0.572	28.52^***^	0.652	808.20^***^
3	SWB	Perceived social support	0.073	5.67^***^	0.456	178.46^***^
		Psychological resilience	0.197	7.68^***^		

*p < 0.05; **p < 0.01; ***p < 0.001.

The regression coefficients were statistically significant (β = 0.572, *p* < 0.001) with psychological resilience as the dependent variable and perceived social support as the independent variable. The regression coefficients for perceived social support (β = 0.073) and psychological resilience (β = 0.197) were also statistically significant (*p* < 0.001) with SWB as the dependent variable and perceived social support and psychological resilience as the independent variables. Perceived social support significantly predicts SWB. When the mediating variable psychological resilience is added, the regression coefficient of perceived social support still has statistical significance, but significantly decreases (the partial regression coefficient decreases from 0.186 to 0.073).

The results imply that psychological resilience has a significant partial mediating effect between perceived social support and SWB, with a mediating effect of 0.113 (0.572 × 0.197), a direct effect of 0.073, and a total effect (mediating effect+direct effect) of 0.186 (0.113 + 0.073). The ratio of mediating effect to total effect is 0.608, and the ratio of direct effect to total effect is 0.392. 39.2 percent of the effects of perceived social support on SWB are direct effects, whilst 60.8 percent are mediated by the mediating variable of psychological resilience (see Figure 1).

## 4 Discussion

The present study discovered a positive correlation between the migrant older adult’s perceived social support, psychological resilience, and SWB. This result is consistent with a number of existing studies on the older adults (38), suggesting a close relationship between perceived social support, psychological resilience, and SWB. Furthermore, it was determined that the migrant older adult’s perceived social support can positively predict SWB. In addition, psychological resilience plays a partial intermediary role in the relationship between perceived social support and SWB. Therefore, the more social support the migrant older adult’s group perceives, the better their psychological resilience. Thus, they will experience higher SWB, consistent with existing research (39–41). Furthermore, existing studies have shown that the social support individuals perceive subjectively can enhance their SWB more than actual social support (38). More specifically, in addition to objective social support, perceived social support is essential in improving individuals’ SWB. This is consistent with the conclusion of the present study.

From the results and effects as verified with the intermediary model, perceived social support directly impacts SWB and indirectly impacts SWB through psychological resilience. The results indicate that the direct effect of perceived social support (39.2 percent) is lower than its indirect effect (60.8 percent). This suggests that the perceived social support influencing SWB through resilience is the primary way perceived social support affects SWB.

Social support is a personal resource that individuals obtain from the outside world. From one perspective, perceived social support indicates the resources individuals receive from social networks. However, in contrast, it reflects the individual’s attitude towards the materials or emotions of the outside world. Therefore, it is closely related to individual personality traits and psychological resources. Furthermore, as a ‘buffer’ against stress, psychological resilience is an essential psychological resource for individuals and is even more closely related to social support. Thus, a social support system can provide individuals with spiritual materials and improve their happiness. Meanwhile, perceived social support can significantly predict psychological resilience (42). Individuals with higher perceived social support own more psychological resources. In the face of threats and pressures, individuals with higher psychological resilience can more effectively use their psychological resources to buffer the negative impact of stress or adversity on their mental health. This strength results in more robust psychological adaptability and higher SWB. Therefore, resilience and perceived social support are closely related and play a vital role in improving SWB.

This research offers certain revelations on the practical operation of the migrant older adult’s SWB. The future intervention in the migrant older adult’s group includes the following aspects. Firstly, from the perspective of social support, families and society should provide adequate support to the migrant older adults. For example, adult children should care more for their migrant older adults regarding economic support and emotional care. In addition, communities with resources could set up older adults community activity centres, organise community activities, and promote communication and exchange. This approach would provide an exchange platform for the older adults to establish a new social network. In addition, when supporting the migrant older adults, the strength and quality of the social support are crucial, namely, to be concerned with the feelings of the older adults to enhance their perceived social support, and truly improve their quality of life and SWB. Secondly, from the perspective of psychological resilience, one of the most critical factors in protecting the psychological health of the migrant older adults, it is important to help them to strengthen their psychological resilience, to improve their ability to [cope with/manage] stress and problems, and help them to adapt to the new social environment. In addition, from the perspective of positive psychology, it is critical to tap into the migrant older adult’s psychological potential and help them construct positive individual experiences, improve their positive psychological qualities and care about the harmonious development of their mental health.

Based on the perspective of positive psychology, this research conducted an empirical study on the SWB and influencing factors of migrant older adults. However, this research still has limitations. Firstly, due to geographical constraints, the investigation was limited to migrant older adults in Dongguan without comparison with those living in other cities. The researchers intend to expand the sampling areas in future research and compare the migrant older adults with other older adults groups to obtain more comprehensive data on the mental health of the migrant older adults. Secondly, the questionnaire in this research was administered in a question-and-report format. Thus, the older adults may differ from one individual to the next in the interpretation of and response to the questions, resulting in deviation in the reports. Therefore, future research could use more effective measurement methods to reduce this error.

In contrast to existing studies, the present study explored the impact of perceived social support and psychological resilience on the SWB of the migrant older adults from a psychological perspective. It focused on the importance of the subjective experience of social support and psychological resilience in enhancing the well-being of the trailing older adults. Future studies could focus on the psychological well-being of migrant older adults from multiple perspectives, including emotion and cognition. Additionally, most existing studies focused on the problems faced by the migrant older adults regarding urban adaptation and social security. Thus, future studies could explore the positive impact of the migrant older adults as key members of migrant families and the backup force of their children on their families, and society to help researchers take a more comprehensive perspective of the migrant older adults.

## Data Availability

The raw data supporting the conclusions of this article will be made available by the authors, without undue reservation.
